# The implementation and evaluation of a family‐led novel intervention for delirium prevention and management in adult critically ill patients: A mixed‐methods pilot study

**DOI:** 10.1111/nicc.13210

**Published:** 2024-12-01

**Authors:** Gideon U. Johnson, Amanda Towell‐Barnard, Christopher McLean, Beverley Ewens

**Affiliations:** ^1^ School of Nursing and Midwifery Edith Cowan University Joondalup Australia; ^2^ Chelsea and Westminster Hospital NHS Foundation Trust London UK; ^3^ Florence Nightingale Faculty of Nursing, Midwifery and Palliative Care King's College London London UK; ^4^ Centre for Nursing Research Sir Charles Gairdner Hospital Nedlands Australia; ^5^ School of Health Sciences University of Southampton Southampton UK

**Keywords:** critical care, delirium, family‐led interventions, non‐pharmacological, voice reorientation

## Abstract

**Background:**

Family‐led interventions have been identified as effective in many areas of care including the management of delirium. However, because of the heterogeneity and ambiguity of family‐led interventions, they are not consistently applied within intensive care units. A user‐friendly digital intervention may therefore support consistent family integration into delirium management.

**Aim:**

To explore the feasibility and acceptability of a family member's voice reorientation intervention for delirium prevention and management in an adult intensive care unit.

**Study Design:**

Parallel, convergent mixed‐methods pilot study was conducted in a general adult intensive care unit in the United Kingdom. Thirty participants (15 patients and 15 family members) were enrolled in the study. For the qualitative component, 17 participants (three patients, six family members and eight nurses) contributed to the evaluation.

**Results:**

The median frequency of the family member's voice reorientation intervention was 2.3 times per day (range 3.3), and the median Richmond Agitation‐Sedation Scale score was −1 (range 2.5). Qualitative data revealed seven themes: acceptance of the intervention, communication, delirium awareness, reactions to the intervention, cognitive state, perception of the intensive care unit and psychological well‐being.

**Conclusion:**

Nurses can involve family members in person‐centred care within the intensive care unit. Results from this study indicate that the family member's voice reorientation programme is feasible and acceptable and may be an effective strategy for providing ongoing orientation, reassurance and comfort to critically ill adult patients to prevent or manage delirium. A larger study is needed to evaluate its impact on delirium.

**Relevance for Clinical Practice:**

The family member's voice reorientation intervention offers critical care nurses a feasible, family‐centred approach to support delirium care in the intensive care unit. Integrating this non‐invasive tool into practice may enable nurses to enhance patient outcomes, reduce anxiety and strengthen collaboration between patients, families and health care professionals.

**What is known about the topic**

- Intensive care unit (ICU) delirium has cognitive, psychological and economic consequences that affect patients, family members, clinical staff and the health care systems.
- Family involvement has been identified as effective in delirium prevention and management.
- There is a lack of standardization of family involvement in delirium practices within ICUs, making it challenging to establish this holistic approach to care

**What this paper adds**

- A family‐led voice reorientation intervention for delirium management in intensive care unit (ICU) is feasible and acceptable and well received by patients, families and clinicians.
- Patients, family members and clinicians' unique perspectives and experiences are valuable in evaluating and refining delirium care interventions.
- A digital family‐led voice reorientation programme can provide nurses with an effective tool to involve family members in patient care and enhance family‐centred care in ICU.

## 1 INTRODUCTION

Delirium occurs in an estimated 33% to 55% of adult patients in intensive care units (ICUs), and is more prevalent in those requiring mechanical ventilation.^1^, ^2^ Delirium prolongs the length of mechanical ventilation, extends hospital length of stay and increases mortality.^3^, ^4^ Delirium is also associated with long‐term cognitive decline, which results in a decreased quality of life.^5^ Delirium can cause significant distress to patients and their families, leading to increased anxiety, depression and impaired psychological well‐being among both groups.^2^ These outcomes result in increased health care costs and increases the disease burden for many individuals.^6^ The mean difference in cost of an ICU stay for patients diagnosed with delirium compared to those who were not was estimated at $3921 (US dollars).^7^ There has been a considerable amount of exploration in the prevention and management of ICU delirium in recent years, which has increased the awareness and reduction in delirium prevalence in ICU.^2^, ^8^ Despite these efforts, substantial work is required to eradicate delirium or minimize the incidence to an insignificant level in ICU.^9^

## 2 BACKGROUND

Delirium prevention and management strategies within ICUs are often pharmacological strategies such as the use of antipsychotic and sedative medications as first‐line approaches.^10^ However, pharmacological strategies alone are not recommended as best practice as their efficacy is limited in this phenomenon and can yield detrimental effects for ICU patients.^11^, ^12^ Antipsychotic medications play no role in the prevention of delirium, and may purely minimize episodes of hyperactive delirium which may result in episodes of hypoactive delirium as a result.^8^, ^11^

There are a variety of non‐pharmacological approaches to the prevention and management of ICU delirium, however, the engagement and utilization of these interventions remains low.^13^ The most common non‐pharmacological interventions in delirium care include cognitive stimulation, environmental orientation, family involvement in patient care, early mobilization and sleep promotion.^8^, ^11^ Barriers to the consistent implementation of non‐pharmacological interventions include the varying nature of the ICU environment, heterogeneity of the patient population, limitations in staff knowledge and skills, resource provision and awareness about the effectiveness of non‐pharmacological interventions in delirium care.^13^, ^14^ There is also a lack of evidence to support the effectiveness of non‐pharmacological interventions utilized over an extensive period as standalone interventions.^15^, ^16^ Non‐pharmacological interventions are often evaluated as part of a multicomponent delirium intervention combining pharmacological and non‐pharmacological interventions, thereby obscuring their efficacy and limitations, limiting the evidence base and opportunities to implement them.^15^

While pharmacological interventions may be important in certain cases, to maintain safety of the patient, there is growing recognition that non‐pharmacological approaches, when designed and implemented effectively, can complement pharmacological treatments by addressing cognitive and emotional aspects of care, especially when tailored to the needs of the patient and family.^17^ One promising strategy to increase engagement and consistent implementation of non‐pharmacological interventions in delirium care is to adopt a straightforward and user‐friendly approach that incorporates the expertise of patients, family members and clinical staff in the design, implementation and evaluation of interventions within delirium care.^8^, ^17^

A promising and evolving area in ICU and delirium care in particular has been the implementation of family involvement. Studies have identified a positive relationship between family involvement and outcomes in delirium care.^18^, ^19^ Active participation in care by family members can be assumed to be possible only if they have a physical presence in ICU, however, this presence can also be achieved virtually. An approach to a virtual family presence could involve the digital delivery of a familiar voice to patients, however, there is a lack of evidence to support the effectiveness of familiar voices on delirium and the patient or family experience. In this study, we explored if a familiar voice digitally delivered to ICU patients may have a positive impact on delirium care, patients' overall well‐being and prevent or minimize anxiety and agitation in mechanically ventilated ICU patients. We applied a co‐design strategy to the development of a family‐led digital intervention, the family member's voice reorientation (FAMVR) intervention that digitally records familiar voices to provide auditory orientation to mechanically ventilated critically ill adult patients in ICU.^20^

## 3 AIMS AND OBJECTIVES

The aims of this mixed‐methods pilot study were: (1) To determine the effect of the FAMVR on Richmond Agitation‐Sedation Scale (RASS) scores^21^ and (2) to explore the experiences of patients, family members and clinical staff to determine the feasibility and acceptability of the FAMVR intervention. These aims were designed to enable the refinement of the FAMVR intervention and the methods and processes in anticipation of conducting a full‐scale randomized controlled trial (RCT).

## 4 DESIGNS AND METHODS

This study is the second phase of a two‐phase study; phase one focused on the development of the FAMVR intervention.^22^ During phase two, a parallel convergent mixed‐methods design^23^ was used. Quantitative and qualitative data were collected from patients, while qualitative data were gathered from family members and nurses. Although data collection involved different participant groups, both types of data were collected concurrently over 6 months and analysed separately, with the integration occurring during the interpretation phase. Integration of both the quantitative and qualitative data occurred 1 month after the completion of the study.^23^ This mixed‐methods approach provided an overview of the effectiveness and limitations of the intervention from the quantitative data, and provided an in‐depth understanding of participants' experiences and perspectives of the family‐led intervention.^23^ Reporting adhered to the CONSORT 2010 statement: extension to randomized pilot and feasibility trials.^24^

### 4.1 Setting

The study was conducted in a 32‐bed medical and surgical adult ICU situated across two sites in one city in the United Kingdom. The ICU is part of the Critical Care Network, which provides care for a diverse population of 2 million people. It is managed under the UK National Health Service (NHS), which provides all patients with access to free health care.

### 4.2 Patient and family member participants and recruitment

#### 4.2.1 Patient participants

Inclusion criteria were patients admitted in ICU, aged 18 years and over, mechanically ventilated with an ICU length of stay ≥24 h and RASS score of ≥−3 at the time of recruitment.

#### 4.2.2 Family participants

Inclusion criteria were participants aged 18 years and over, and willingness to participate in a one‐to‐one interview with the researcher after the patient was discharged from the hospital. Additional inclusion criteria for family member participants were: willingness to record their voices on an iPad, understand English, have a family member in the ICU and provide written informed consent.

Patients who were sedated and mechanically ventilated were deemed to lack the mental capacity to provide informed consent for a research study.^25^, ^26^ Therefore, an appropriate consultee who was known to the patient as a family member was required to provide advice about enrolling eligible patients for the research study, and when the patient regained capacity, an informed decision could then be obtained. If patients did not provide written informed consent, their data were not used for the study. Exclusion criteria for patients comprised those who were receiving palliative care or at the end of life.

Potential participants who met the inclusion criteria were identified via the hospital's Electronic Medical Record (EMR). The researcher then visited the ICU during visiting hours to assess patients' eligibility and provide information about the study to family members who were present. A recruitment flyer which included the researcher's contact number and email address, participant information form (PIF) and consultee information sheet (CIS) were given to family members. Family members interested in participating in the study contacted the researcher by telephone and were screened at the same time to ensure they met the inclusion criteria. The researcher then arranged a mutually convenient time to meet to obtain written informed consent from the family member participants and who provided a consultee declaration on behalf of the patient participants. The recording for the FAMVR intervention was then conducted during this meeting.

### 4.3 Nurse participants recruitment

Inclusion criteria for nurse participants were nurses employed permanently in clinical positions during the study period, had ≥6 months ICU experience, willing to participate in a one‐to‐one or small group education session before using the intervention, willing to participate in a focus group discussion and ability to provide written informed consent.

Support was provided by the ICU clinical nurse educator at the study site, who shared an electronic recruitment flyer with all nursing staff. The flyer comprised information about the study and the researcher's contact number and email address. The researcher also visited the ICU during team meetings to provide information about the study, and collected contact details of potential participants. Some potential participants contacted the researcher, and the researcher contacted those who provided their contact details. The researcher provided the PIF to the participants and obtained written informed consent at this time.

### 4.4 Intervention

The FAMVR intervention is a family member's recorded messages on an iPad, co‐designed with people with lived experience of ICU as patients, family members and nursing and medical professionals.^22^ This development process was iterative where the co‐designers' experiences and knowledge contributed to the development of the intervention prototype (McAllister et al., 2021;^27^, ^28^). An evidence‐based design model, the Double Diamond,^29^ was utilized to ensure power was equally shared among the participants in the creation process. The methodical approach, guided by the Double Diamond model ensured rigour to the development processes of the intervention.^18^

The FAMVR intervention comprises four domains (see Supplementary materials: the FAMVR intervention messages). The first domain is the ICU general reorientation domain, which consists of orientation messages about the ICU environment as well as providing reassuring messages to patients. The second domain was the ICU routine domain, which comprises messages which provided information to patients before procedures such as washing or repositioning. The third domain was the ICU procedure domain, where the messages provided orientation about the endotracheal tube during spontaneous breathing trials and ventilation weaning. The fourth domain was the free category, which the family members who were not visiting regularly could contribute by providing context of everyday family events for the patient. The first domain, general orientation, was designed to be played three times a day (morning, afternoon and night), whereas the other domains were designed to be played when required, that is, before care delivery or procedures. Each domain consisted of 10 standardized messages, with the total recording time for each domain lasting 1 to 3 min. Family members were provided with a structured template and clear instructions for recording messages related to orientation, reassurance and personal memories. A research team member guided the process to ensure consistency and adherence to the protocol.

Four training sessions were held with nurse participants in order to refresh their knowledge of delirium, RASS score assessments and to explain the FAMVR intervention. The RASS^30^ was used to assess patients' sedation and agitation levels during the FAMVR intervention. The RASS, a validated and widely utilized tool in ICU settings, ranges from +4 (combative) to −5 (unarousable), with a score of 0 representing alert and calm, the clinical target for most ICU patients.^30^ Scores of 0 to −2 are considered optimal for lightly sedated patients, enabling adequate responsiveness. The RASS has demonstrated strong inter‐rater reliability and validity in critically ill patients, making it an appropriate choice for monitoring agitation‐sedation in this study.^21^

The RASS score was used in preferred to the confusion assessment method for the intensive care unit (CAM‐ICU)^30^ as RASS is assessed hourly for every patient, and if an adverse reaction occurred during the implementation of the FAMVR intervention, this would have been quickly identified, and the intervention discontinued. The nursing staff had good knowledge and compliance with the RASS score measurement compared to the CAM‐ICU during the education sessions and from audit compliance of the ICU. A total of 22 nurses attended the education sessions about the FAMVR intervention, and several one‐to‐one sessions were conducted at the bedside until all nursing staff in the ICU had received the education. During the implementation of the FAMVR intervention, bedside education continued with nurses and family members; feedback about the intervention was obtained as part of the refinement process.

Nurse participants were instructed to discontinue the intervention if a patient showed an adverse reaction or sudden increase in RASS score of 2 and above (see Supplementary material, RASS), and guidance was provided to the nurses on what actions to take should this occur. The intervention process consisted of playing the FAMVR to patients, and collecting data on the message domains, frequency delivered and any sedatives currently being administered to the patient. The researcher visited enrolled patient participants at least once daily to provide further guidance to nurse and family participants, clarify any questions, perform RASS assessments and check the medication charts to ensure they corresponded with what had been documented. Patient participants' treating physicians were notified about their participation in the study via documentation in the EMR.

### 4.5 Primary outcome measures: Richmond Agitation‐Sedation Scale score

The primary outcome was the number of patient participants who achieved a decrease in RASS score and no increase in RASS score during the implementation of the FAMVR intervention. The primary outcome was assessed at the same time daily for each patient participant until they were no longer mechanically ventilated. The daily median RASS scores were calculated for each patient participant on the days that the FAMVR intervention was played to patients.

### 4.6 Secondary outcomes: Intervention feasibility and acceptability

Exploring the feasibility and acceptability included interest in the study, retention rates of patient and family member participants, recruitment processes, patient and family participation rates in the interviews after discharge from the hospital and data collection and analysis processes. The experiences and perceptions of nurses about the FAMVR were also explored via a focus group to determine the feasibility and acceptability of the intervention.

### 4.7 Sample size

The sample size was estimated based on the recommendation for pilot studies,^31^, ^32^ and designed to be both practical and appropriate to ensure that sufficient data were collected to inform future larger‐scale studies. A sample size of 30 participants, 15 patients and 15 family members were selected for the study. Nursing participants were included in addition to ensure sufficient data were gathered about the acceptability of the intervention. Within the qualitative component, we aimed for 15 patient/family interviews and 2–3 focus groups with nurses, to achieve data saturation to ensure we had captured all of the participants' experiences with the FAMVR intervention while maintaining feasibility of the intervention within the clinical setting.

### 4.8 Data collection and analysis

#### 4.8.1 Quantitative data

Data were collected via patient records which included the number of times the intervention was played and the patients' sedation statuses at the time the interventions were played. The RASS score, types of sedatives and patient demographics were obtained from the EMR. Descriptive statistics were used for the quantitative component of the study, including the RASS score measurement, patient participant demographics and the frequency at which the FAMVR was played to patient participants. Given the small sample size and non‐normal data distribution, Microsoft Excel was used to conduct the statistical analysis to determine the median and the range of the data.^33^

#### 4.8.2 Qualitative data

Qualitative data were captured via a combination of semi‐structured interviews with patient and family member participants, and a virtual focus group with nurses until data saturation was achieved. Semi‐structured interviews and focus groups were digitally audio‐recorded and transcribed verbatim via NVivo transcription software^34^ (see Supplementary materials: semi‐structured interview questions and focus group interview questions). Interview transcripts were imported into NVivo qualitative analysis software, where the transcribed interviews were read and coded and agreed upon with the research team. Thematic analysis was used to analyse the interviews where initial codes were generated to reflect the key topics identified in the interviews and the themes that emerged from the codes.^35^ To ensure trustworthiness in the qualitative component of the study, we adhered to the established criteria of credibility, dependability and confirmability by using member checking, triangulation of data sources and maintaining an audit trail throughout the analysis process.^36^

#### 4.8.3 Integrated analysis

The quantitative and qualitative data integration^37^ occurred within the month after the study period using data triangulation, with both datasets analysed in parallel to cross‐validate and reinforce the findings from each method, ensuring trustworthiness through comparison. Quantitative data, such as RASS scores and frequency of the FAMVR intervention, were merged with qualitative data about participants' experiences to provide an in‐depth understanding of the facilitators and barriers that might have influenced the intervention's efficacy, feasibility and acceptability.

### 4.9 Ethical and research approval

Ethical approval was obtained on 7 March 2023 from the Health Research Authority (HRA) and Health and Care Research Wales (HCRW), and Research Ethics Committee (ref: 23/LO/0057) at the study site. Reciprocal approval was received on 28 March 2023 from the university Human Research Ethics Committee (REMS NO: 2023‐04186‐JOHNSON) where the author is a doctoral candidate. The study was prospectively registered on the Australian New Zealand Clinical Trials Registry (ANZCTR); trial ID: ACTRN12622001568707; ANZCTR ‐ Registration.

## 5 FINDINGS

### 5.1 Intervention enrolment

**FIGURE 1:**
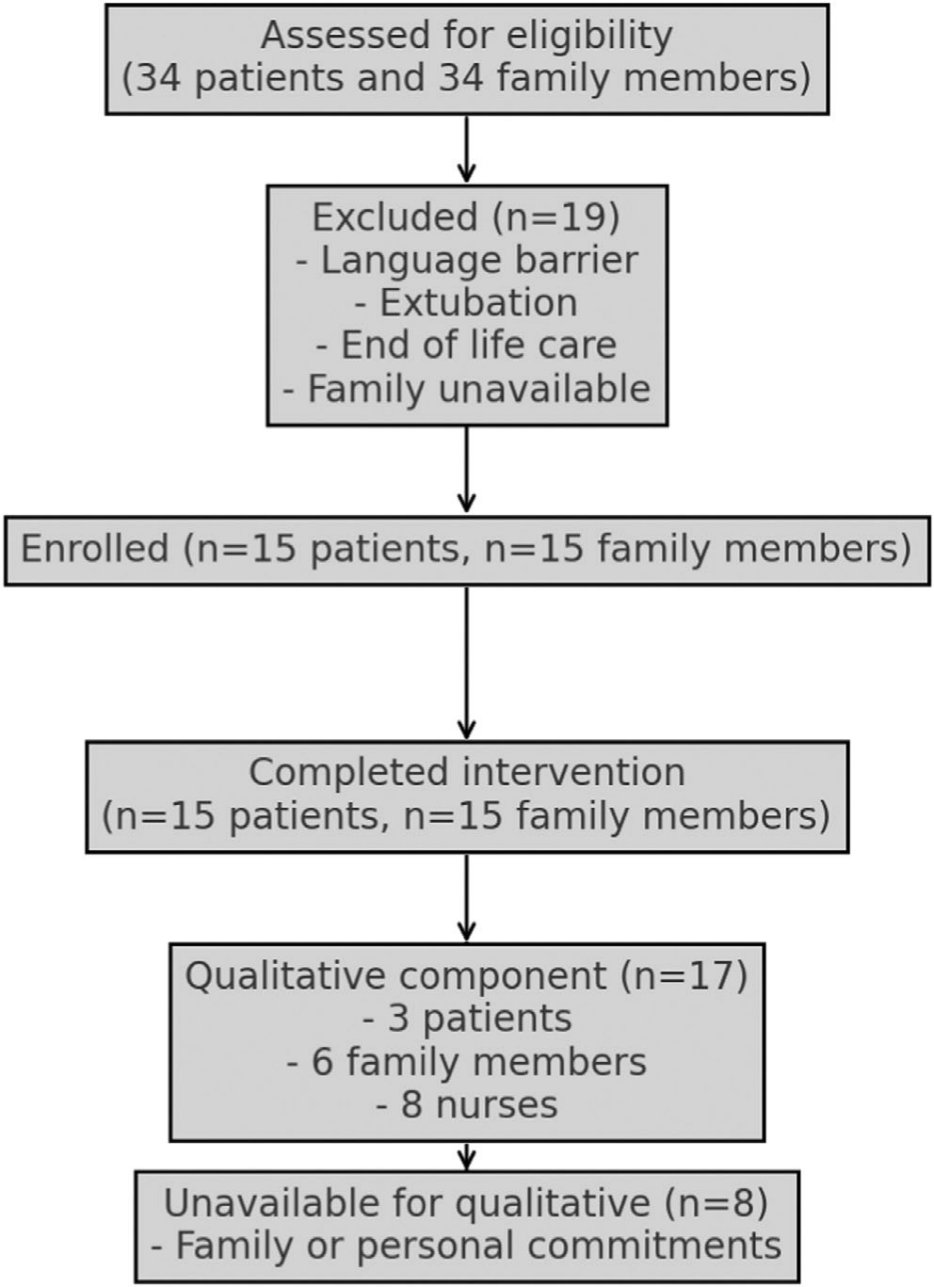
Flow of participants in the nonrandomized pilot study.

A total of 30 participants (patients *n* = 15, family *n* = 15) were enrolled in the study. The patient participants (*n* = 15) received the FAMVR intervention, and the family member participants (*n* = 15) recorded the messages in the FAMVR intervention. The target enrolment of 30 participants was achieved within 6 months of the intervention period (September 2023–February 2024). Retention in the study was 100% throughout the study period, as no family member withdrew their advice to continue enrolling patients (Figure 1). Nine participants (patients *n* = 3; family *n* = 6) participated in the one‐to‐one semi‐structured interviews after discharge from the hospital and eight nursing staff agreed to participate in a focus group.

### 5.2 Patient participants' characteristics

Patient demographic data, ventilation mode, sedation status and psychiatric history were collected at baseline (Table 1). The median age of participants was 69 years (range: 46), and the median number of days on mechanical ventilation was 5 (range: 1–31). Most participants were female (*n* = 8) and all spoke English as their first language (*n* = 13). At baseline, the majority were mechanically ventilated via an endotracheal tube (ETT) (*n* = 14) and sedated (*n* = 10). Most patients (*n* = 10) were ventilated for 7 days or fewer following the commencement of the intervention. Diagnoses ranged across sepsis, respiratory failure and cardiac arrest; however, individual diagnoses were not specifically collected as they were not critical to the focus of the pilot study. Importantly, no participants had psychiatric or neurological conditions that might directly impact RASS scores or the intervention.

**TABLE 1:** Patient participants' baseline characteristics.

Characteristics	All participants (*n* = 15)	Semi‐structured interview participants (*n* = 3)
Median age (years), *n* (Range)	69 (46)	70 (23)
Male, *n* (%)	7 (46.7%)	2 (66.7%)
Female, *n* (%)	8 (53.3%)	1 (33.3%)
English as first language, *n* (%)	13 (86.7%)	3 (100%)
English as second or third language *n* (%)	2 (13.3%)	0 (0%)
Married/partnered, *n* (%)	9 (60%)	3 (100%)
Median ventilation (days), *n* (Range)	5 (31)	3 (2)
Sedated, *n* (%)	10 (66.7%)	1 (33.3%)
Not sedated, *n* (%)	5 (33.3%)	2 (66.7%)
Psychiatric diagnosis	0 (0%)	0 (0%)

Abbreviation: n, number.

### 5.3 Family participants' characteristics

Demographics and family members' relationship to the patients were captured before the start of the intervention (Table 2). Most family participants were female (*n* = 12), and most participants had English as their first language (*n* = 12). Most of the participants were spouses or partners to the patient participants (*n* = 7) and lived with them.

**TABLE 2:** Family participants' characteristics.

Characteristics	All participants (*n* = 15)	Semi‐structured interview participants (*n* = 6)
Male, *n* (%)	3 (20%)	3 (50%)
Female, *n* (%)	12 (80%)	3 (50%)
English as first language, *n* (%)	12 (80%)	5 (83.3%)
English as a second or third language, *n* (%)	3 (20%)	1 (16.7%)
Spouse/Partner	7 (46.7%)	3 (50%)
Other relatives, for example, son, daughter, sibling, cousin, grandchild	8 (53.3%)	3 (50%)
Living with patient	7 (46.7%)	3 (50%)
Not living with the patient	8 (53.3%)	3 (50%)

Abbreviation: n, number.

### 5.4 Quantitative results

**FIGURE 2:**
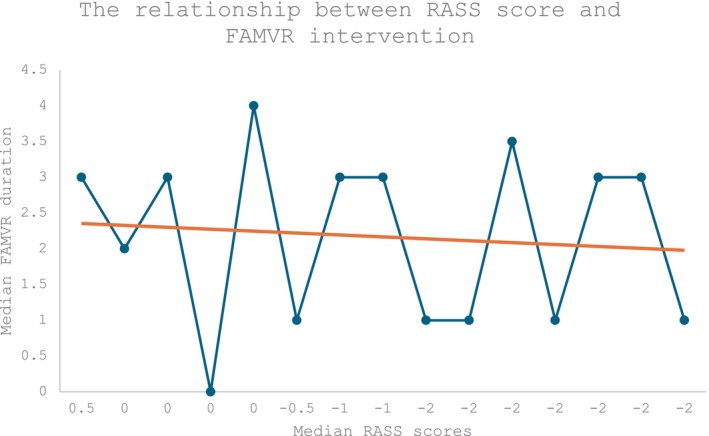
The relationship between Richmond Agitation‐Sedation Scale score and Family Member's Voice Reorientation intervention among all patient participants (*n* = 15).

Descriptive statistics were used to analyse the quantitative data in Microsoft Excel. Table 3 outlines the median and range of RASS scores and FAMVR intervention frequency for patient participants. The median RASS score during the study was −1 (range: −2 to 0), and the median frequency of the FAMVR intervention was 2.3 times per day (range: 1–4). Figure 2 illustrates the relationship observed between median RASS outcomes and FAMVR frequency for all patients (*n* = 15). All participants completed the intervention, and no adverse reactions were reported (adverse reaction = 0), leading to a 100% retention rate. Most patients (*n* = 12) received the intervention at least three times per day, and all (*n* = 15) received it at least once daily. Five participants achieved a RASS score of 0 (alert and calm), and three patients improved from +1 (restlessness) to 0 by the end of the study.

**TABLE 3:** Median and range of Richmond Agitation‐Sedation Scale scores and Family Member's Voice Reorientation frequency among participants.

RASS score	Number of patients (*n*)	Median RASS	Range RASS	Median FAMVR	Range FAMVR
RASS 0 to −1 (normal range)	8	0	0 to −1	2.5	1–4
RASS −2 (light sedation)	7	−2	−2 to −2	3	1–4
Total	15	−1	0 to −2	2.3	1–4

### 5.5 Qualitative analysis: Participants' experiences with the intervention

Three patient participants and six family partipants were interviewed during this study phase. Of the eight nursing staff who participated in the focus group, four were senior ICU nurses (≥5 years experience), and four junior ICU nurses (≤5 years experience). Two of the nursing staff were male, and six were female. Tables 1 and 2 outline the characteristics of the patient and family participants. The interview data from patient and family member participants were analysed separately from the focus group data. Thematic analysis of both datasets identified seven key themes: acceptance of the FAMVR intervention, communication processes, delirium awareness, reactions to FAMVR, cognitive state, perception of the ICU and psychological well‐being (Table 4).

**TABLE 4:** Key themes from qualitative analysis, stratified by participant group (patients, family members and nurses).

Themes	Explanation	Representative quotes
Acceptance of FAMVR	Participants found the FAMVR intervention feasible and valuable for involving family members in patient care, particularly outside of visiting hours, enhancing overall care.	‘We really want the family to be involved in their care… So really do find it very handy’. {Nurse 2}
Communication	The intervention facilitated good communication between patients, family members and health care professionals, contributing to positive perceptions of the ICU experience.	‘Everyone was so nice and caring… And then it was as good as I imagined it could be’. {Family member of patient 8}
Delirium awareness	Nurses noted that the intervention increased their knowledge of delirium, although some still expressed uncertainty in identifying it. There was a call for more education to improve understanding and use of the intervention.	‘Because sometimes I'm not really sure if the patient is… delirious or just confused or in pain’. {Nurse 4} ‘It has increased my knowledge on delirium…’ {Nurse 1}
Reactions to FAMVR	While some family members found recording messages emotionally challenging, they ultimately perceived the intervention as beneficial, offering reassurance and comfort.	‘It was a bit weird, not detrimentally’. {Family 2} ‘I found that the fact that you would ask me to do that, is quite moving in itself’. {Family 4}
Cognitive state	The FAMVR intervention was reported by nurses to improve patients' alertness and cognitive function, contributing to reduced sedation and quicker weaning from mechanical ventilation.	‘It actually helps with the delirious process… patients are feasible to come off the sedation’. {Nurse 6}
Perceptions of the ICU	The intervention supported person‐centred care and reassured family members, but nurses highlighted challenges in documenting the intervention on electronic medical records (EMR), suggesting FAMVR should be integrated into the system.	‘If it's more on CERNER and we put it like a part of our daily handover, we wouldn't miss it’. {Nurse 1}
Psychological well‐being	Patients generally returned to baseline well‐being post‐intervention, while family members and nurses reported that their own psychological well‐being was not negatively affected.	‘I don't feel like affected at all. I just know this is for the benefit of my patients’. {Nurse 2}

### 5.6 Integrated results with data triangulation

The integration of the quantitative data (RASS scores and FAMVR frequency) with the qualitative themes provides a comprehensive understanding of the FAMVR intervention's impact on patients, families and nurses.

#### 5.6.1 Acceptance of FAMVR

Patients with RASS scores of −2 to −1, such as Patient 11 (median RASS = −2, FAMVR = 3.5), showed strong acceptance of the intervention. A nurse noted, ‘When they hear from their loved one's voice, it's more factual, and they take it easily’ (Nurse 1). Similarly, family members, like the relative of Patient 11, valued the intervention, stating, ‘I knew that it would go maybe into the subconscious’.

#### 5.6.2 Communication

Both patients and family members expressed positive feelings about communication during the intervention. Patient 11 shared, ‘She understood me… she was the most amazing nurse’, while the family member of Patient 8 (median RASS = −1, FAMVR = 3) noted the high level of care, saying, ‘Everybody was paying really good attention to his case’.

#### 5.6.3 Delirium awareness

Awareness about delirium varied. Some family members were informed, like the relative of Patient 9 (median RASS = −2, FAMVR = 1), who said, ‘I was warned about the possibility of delirium’, while others were not, as noted by the family member of Patient 7, ‘Nobody gave me any information about anything like that’.

#### 5.6.4 Reactions to FAMVR

Emotional reactions were common, with Patient 7 (median RASS =.−1, FAMVR = 3) stating, ‘I was calm. Very happy to have your own care’. Nurses observed responses like tears and facial expressions, as described by Nurse 1, ‘I've seen some response on them, like tears coming from their eyes’.

#### 5.6.5 Cognitive State

Nurses noted improvements in cognitive function during the intervention. Nurse 6 stated, ‘When the family member is around… it actually helps with the delirious process’. This was especially relevant for patients with lower RASS scores who became more alert.

#### 5.6.6 Perception of the ICU

Family members generally felt positive about the ICU experience. The relative of Patient 13 (median RASS = −2, FAMVR = 3) expressed satisfaction, saying, ‘I felt like you gave him his best chance’.

#### 5.6.7 Psychological well‐being

Post‐discharge, patients like Patient 8 (median RASS = −1, FAMVR = 3) reported stable psychological well‐being, stating, ‘I think my mood hasn't been particularly bad’, while Patient 7 noted, ‘Very good… Sometimes I forget, now and again’.

By integrating quantitative RASS data with qualitative insights, the study shows that FAMVR is well‐accepted and has a positive impact on the communication, cognitive state and emotional well‐being of patients, families and nurses in the ICU.

## 6 DISCUSSION

This pilot study was a preliminary investigation to pilot the FAMVR intervention, a novel digital evidence‐based voice reorientation programme to prevent and manage delirium among critically ill adult patients on mechanical ventilation. It aimed to explore the relationship between the FAMVR intervention and the RASS scores of ICU patients and the acceptability and feasibility of the intervention as preliminary work for a large‐scale RCT. To the best of our knowledge, this is the first mixed‐methods pilot study to test a digital family‐led voice reorientation programme with mechanically ventilated ICU patients. The FAMVR had no negative impact on patients' RASS scores. Confounding variables such as the rate of sedation and comorbidities need to be explored in future studies. The qualitative findings from this pilot study revealed that FAMVR is acceptable and valuable for patients, family members and clinicians. The findings also indicate that there is an opportunity for improvement and refinement of the FAMVR based on suggestions from the participants. Achieving the target sample size suggests similar recruitment techniques could be applied for a future RCT or larger‐scale study. The FAMVR was a safe intervention in this study, one that was acceptable and welcomed by patients and families. Further studies are therefore justified in order to refine and develop the intervention and to determine its impact on the incidence and severity of delirium. The FAMVR intervention provides critical care nurses with a family‐centred tool for delirium prevention and management, enhancing communication, patient outcomes and family involvement in the ICU.

The implementation of a digital clinical intervention that is co‐designed with end‐users has shown to be successful and prioritizes end‐users of the intervention, which in turn can lead to the sustainability of such interventions.^38^, ^39^ However, challenges exist when implementing such interventions,^27^, ^39^ as the intricacies of the ICU environment can compound these challenges.^40^ The digital form of the FAMVR programme proved a challenge to some nursing staff while implementing the intervention. Also, the virtual engagement of participants during the evaluation process proved challenging to those who were not proficient in the use of technology. These are common challenges associated with the co‐design approach and technology integration in ICUs.^40^, ^41^ Recognizing these constraints was essential in ensuring the optimal engagement of participants at the co‐design and implementation phases (McAllister et al., 2021;^28^, ^42^). These challenges can be mitigated by simplifying the engagement process and providing accessible support and education to patients, families and clinical staff, which can enable the transferability of the FAMVR intervention to other aspects of ICU care, such as patient diaries and ICU follow‐up clinics.^43^, ^44^ The FAMVR has potential to be applied to other populations including paediatrics and adult populations in ICU follow‐up clinics to improve the quality of life for ICU survivors.^45^, ^46^ Patients and families can revisit their experiences, gain support and access resources upon discharge from the hospital using a digital app. This aspect of care will be explored in future studies.

Our findings in relation to acceptability of the FAMVR intervention align with other studies^47^ which reported positive patient outcomes such as anxiety, satisfaction and health‐related quality of life with family‐centred interventions. The gap in delirium awareness by family members is consistent with other studies.^48^ This study revealed that family members are willing to be involved in delirium care, but the lack of knowledge and information about delirium inhibits their involvement. Interventions such as the FAMVR can promote family members' involvement in delirium care and improve their awareness of this phenomenon. Family‐centred care is an emerging area in ICU, but there are inconsistencies in the implementation of family‐centred care practices.^49^ Family involvement requires a time commitment from staff, staff resources and flexible visitation policies, which may not be feasible in some ICUs.^49^ The environmental design of the ICU and organizational barriers may further impede family involvement. Designing and implementing evidence‐based and consistent family‐centred care policies is essential to manage these challenges, and should be explored further in future studies.

### 6.1 Strengths and limitations

This pilot study provided opportunities to implement a novel family‐led intervention for delirium involving patients, family members and clinicians. Conducting research with a vulnerable population such as those in ICU and patients who lack mental capacity is challenging and takes considerable resources and time. Therefore, while this is a small‐scale study, we have contributed towards the body of knowledge and provided preliminary results and findings which are promising. This work will provide a foundation upon which larger‐scale studies can be based.

Although the sample size in this study was appropriate for a pilot study, it was small and which reduces the generalizability of the findings. The study was undertaken in one study site, which also reduces the generalizability of the findings to the wider ICU community. While four training sessions were held, the training was not standardized across all nurses. Some nurses received bedside one‐to‐one education during the intervention period, which may have led to variations in understanding and implementation of the FAMVR intervention.

The outcomes determined from the RASS score measurement may be valuable in assisting us with the primary outcome variables of an RCT study. Of the 15 patient participants enrolled in the intervention, only three agreed to be interviewed at the 3‐month point after hospital discharge, and does not reflect the experiences of all patient participants' experiences in this study.

## 7 IMPLICATIONS FOR PRACTICE AND CONCLUSION

A simplified and standardized digital family‐led voice orientation intervention can be developed, implemented and evaluated successfully in adult ICUs collaboratively with stakeholders. The FAMVR intervention can positively affect patients' RASS scores and was considered valuable by patients, family members and clinicians. The FAMVR programme is acceptable to ICU delirium management and feasible to implement into clinical practice. The approach can be adopted for a larger‐scale study whereby its effectiveness on delirium outcomes can be substantially tested.

## AUTHOR CONTRIBUTIONS

Gideon U. Johnson: Conceptualization, Methodology, Software, Validation, Formal analysis, Investigation, Resources, Data curation, Writing—original draft, Writing—review and editing, Visualization, Project administration. Amanda Towell‐Barnard: Conceptualization, Methodology, Validation, Formal analysis, Resources, Writing—review and editing, Supervision. Christopher McLean: Methodology, Validation, Formal analysis, Resources, Writing—review and editing, Supervision. Beverley Ewens: Conceptualization, Methodology, Validation, Formal analysis, Resources, Writing—review and editing, Supervision.

## FUNDING INFORMATION

This research was supported by an Australian Government Research Training Program (RTP) Scholarship and a Higher Degree by Research Scholarship and Stipend awarded by Edith Cowan University, WA, Australia.

## CONFLICT OF INTEREST STATEMENT

The authors declare no conflict of interest.

## Supporting information


**Data S1.** Supporting information.

## Data Availability

The data that support the findings of this study are available on request from the corresponding author. The data are not publicly available due to privacy or ethical restrictions.

## References

[1] Pun BT , Badenes R , Heras La Calle G , et al. Prevalence and risk factors for delirium in critically ill patients with COVID‐19 (COVID‐D): a multicentre cohort study. Lancet Respir Med. 2021;9(3):239‐250. doi:10.1016/S2213-2600(20)30552-X 33428871 PMC7832119

[2] Wu N , Zhang B , Wang Y , Zhao H , Zhong M . Incidence, prevalence and risk factors of delirium in ICU patients: a systematic review and meta‐analysis. Nurs Crit Care. 2022;28(5):653‐669. doi:10.1111/nicc.12857

[3] Alzoubi E , Shaheen F , Yousef K . Delirium incidence, predictors and outcomes in the intensive care unit: a prospective cohort study. Int J Nurs Pract. 2023;30(1):1‐7. doi:10.1111/ijn.13154 37044382

[4] Salluh JI , Wang H , Schneider EB , et al. Outcome of delirium in critically ill patients: systematic review and meta‐analysis. BMJ (Clinical Research Ed). 2015;350:h2538. doi:10.1136/bmj.h2538 PMC4454920 26041151

[5] Wilcox ME , Girard TD , Hough CL . Delirium and long term cognition in critically ill patients. BMJ (Clinical Research Ed). 2021;373:n1007. doi:10.1136/bmj.n1007 34103334

[6] Kinchin I , Mitchell E , Agar M , Trépel D . The economic cost of delirium: a systematic review and quality assessment. Alzheimers Dement. 2021;17(6):1026‐1041. doi:10.1002/alz.12262 33480183

[7] Dziegielewski C , Skead C , Canturk T , et al. Delirium and associated length of stay and costs in critically ill patients. Crit Care Res Prac. 2021;2021:6612187. doi:10.1155/2021/6612187 8. PMC8088381 33981458

[8] Mart MF , Williams Roberson S , Salas B , Pandharipande PP , Ely EW . Prevention and management of delirium in the intensive care unit. Semin Respir Crit Care Med. 2021;42(1):112‐126. doi:10.1055/s-0040-1710572 32746469 PMC7855536

[9] Kotfis K , van Diem‐Zaal I , Williams Roberson S , et al. The future of intensive care: delirium should no longer be an issue. Crit Care. 2022;26(1):200. doi:10.1186/s13054-022-04077-y 35790979 PMC9254432

[10] Barbateskovic M , Krauss SR , Collet MO , et al. Pharmacological interventions for prevention and management of delirium in intensive care patients: a systematic overview of reviews and meta‐analyses. BMJ Open. 2019;9(2):e024562. doi:10.1136/bmjopen-2018-024562 PMC6377549 30782910

[11] Chen TJ , Traynor V , Wang AY , et al. Comparative effectiveness of non‐pharmacological interventions for preventing delirium in critically ill adults: a systematic review and network meta‐analysis. Int J Nurs Stud. 2022;131:104239. doi:10.1016/j.ijnurstu.2022.104239 35468538

[12] Tran A , Blinder H , Hutton B , English SW . A systematic review of Alpha‐2 agonists for sedation in mechanically ventilated neurocritical care patients. Neurocrit Care. 2018;28(1):12‐25. doi:10.1007/s12028-017-0388-5 28547318

[13] Lange S , Mędrzycka‐Dąbrowska W , Friganovic A , Oomen B , Krupa S . Non‐pharmacological nursing interventions to prevent delirium in ICU patients‐an umbrella review with implications for evidence‐based practice. J Pers Med. 2022;12(5):760. doi:10.3390/jpm12050760 35629183 PMC9143487

[14] Kang J , Lee M , Ko H , et al. Effect of nonpharmacological interventions for the prevention of delirium in the intensive care unit: a systematic review and meta‐analysis. J Crit Care. 2018;48:372‐384. doi:10.1016/j.jcrc.2018.09.032 30300863

[15] Deng LX , Cao L , Zhang LN , Peng XB , Zhang L . Non‐pharmacological interventions to reduce the incidence and duration of delirium in critically ill patients: a systematic review and network meta‐analysis. J Crit Care. 2020;60:241‐248. doi:10.1016/j.jcrc.2020.08.019 32919363

[16] Luther R , McLeod A . The effect of chronotherapy on delirium in critical care – a systematic review. Nurs Crit Care. 2018;23(6):283‐290. doi:10.1111/nicc.12300 28508438

[17] Liang S , Chau JPC , Lo SHS , Zhao J , Liu W . Non‐pharmacological delirium prevention practices among critical care nurses: a qualitative study. BMC Nurs. 2022;21(1):235. doi:10.1186/s12912-022-01019-5 36008783 PMC9404567

[18] Johnson GU , Towell‐Barnard A , McLean C , Robert G , Ewens B . Co‐designing a digital family‐led intervention for delirium prevention and management in adult critically ill patients: an application of the double diamond design process. Int J Nurs Stud. 2024;160:104888. doi:10.1016/j.ijnurstu.2024.104888 39303642

[19] Qin M , Gao Y , Guo S , Lu X , Zhu H , Li Y . Family intervention for delirium for patients in the intensive care unit: a systematic meta‐analysis. J Clin Neurosci. 2022;96:114‐119. doi:10.1016/j.jocn.2021.11.011 34838428

[20] Johnson GU , Towell‐Barnard A , McLean C , Ewens B . Delirium prevention and management in an adult intensive care unit through evidence‐based non‐pharmacological interventions: a scoping review. Collegian. 2024a;31(4):232‐251. doi:10.1016/j.colegn.2024.05.001

[21] Ely EW , Truman B , Shintani A , et al. Monitoring sedation status over time in ICU patients: reliability and validity of the Richmond agitation‐sedation scale (RASS). JAMA. 2003;289(22):2983‐2991. doi:10.1001/jama.289.22.2983 12799407

[22] Johnson GU , Towell‐Barnard A , McLean C , Ewens B . The development of a family‐led novel intervention for delirium prevention and management in the adult intensive care unit: A co‐design qualitative study. Aust Crit Care. 2024;101088. doi:10.1016/j.aucc.2024.07.076 39129064

[23] Schoonenboom J , Johnson RB . How to construct a mixed methods research design. Kolner Z Soz Sozpsychol. 2017;69(Suppl 2):107‐131. doi:10.1007/s11577-017-0454-1 28989188 PMC5602001

[24] Eldridge SM , Chan CL , Campbell MJ , et al. CONSORT 2010 statement: extension to randomised pilot and feasibility trials. Pilot Feasibility Stud. 2016;2:64. doi:10.1186/s40814-016-0105-8 27965879 PMC5154046

[25] Ecarnot F , Quenot JP , Besch G , Piton G . Ethical challenges involved in obtaining consent for research from patients hospitalized in the intensive care unit. Ann Transl Med. 2017;5(Suppl 4):S41. doi:10.21037/atm.2017.04.42 29302597 PMC5750252

[26] Mental Capacity Act 2005 . (2005). *Legislation.gov.uk*. http://www.legislation.gov.uk/ukpga/2005/9/contents (Accessed June 3, 2024).

[27] Raynor DK , Ismail H , Blenkinsopp A , Fylan B , Armitage G , Silcock J . Experience‐based co‐design‐adapting the method for a researcher‐initiated study in a multi‐site setting. Health Expect. 2020;23(3):562‐570. doi:10.1111/hex.13028 32045087 PMC7321746

[28] Slattery P , Saeri AK , Bragge P . Research co‐design in health: a rapid overview of reviews. Health Res Policy Sys. 2020;18:17. doi:10.1186/s12961-020-0528-9 PMC7014755 32046728

[29] Design Council UK . The Design Process: What Is the DoubleDiamond?. Design Council; 2019.

[30] Ely EW , Inouye SK , Bernard GR , et al. Delirium in mechanically ventilated patients: validity and reliability of the confusion assessment method for the intensive care unit (CAM‐ICU). JAMA. 2001;286(21):2703‐2710. doi:10.1001/jama.286.21.2703 11730446

[31] Hertzog MA . Considerations in determining sample size for pilot studies. Res Nurs Health. 2008;31(2):180‐191. doi:10.1002/nur.20247 18183564

[32] Teresi JA , Yu X , Stewart AL , Hays RD . Guidelines for designing and evaluating feasibility pilot studies. Med Care. 2022;60(1):95‐103. doi:10.1097/MLR.0000000000001664 34812790 PMC8849521

[33] Cooksey RW . Descriptive statistics for Summarising data. Illustrating Statistical Procedures: Finding Meaning in Quantitative Data. Springer; 2020:61‐139. doi:10.1007/978-981-15-2537-7_5

[34] Lumivero . *NVivo* (Version 14). 2023 www.lumivero.com

[35] Braun V , Clarke V . Using thematic analysis in psychology. Qual Res Psychol. 2006;3(2):77‐101. doi:10.1191/1478088706qp063oa

[36] Lincoln Y , Guba EG . Naturalistic Inquiry. Vol 9. SAGE Publications; 1985:438‐439. doi:10.1016/0147-1767(85)90062-8

[37] Fetters MD , Curry LA , Creswell JW . Achieving integration in mixed methods designs‐principles and practices. Health Serv Res. 2013;48(6 Pt 2):2134‐2156. doi:10.1111/1475-6773.12117 24279835 PMC4097839

[38] Bird M , McGillion M , Chambers EM , et al. A generative co‐design framework for healthcare innovation: development and application of an end‐user engagement framework. Res Involv Engagem. 2021;7(1):12. doi:10.1186/s40900-021-00252-7 33648588 PMC7923456

[39] Istanboulian L , Rose L , Yunusova Y , Dale C . Adapting co‐design methodology to a virtual environment: co‐designing a communication intervention for adult patients in critical care. Res Involv Engagem. 2023;9(1):103. doi:10.1186/s40900-023-00514-6 37957776 PMC10644625

[40] Meissen H , Gong MN , Wong AI , et al. The future of critical care: optimizing technologies and a learning healthcare system to potentiate a more humanistic approach to critical care. Crit Care Explor. 2022;4(3):e0659. doi:10.1097/CCE.0000000000000659 35308462 PMC8926065

[41] Kirk J , Bandholm T , Andersen O , et al. Challenges in co‐designing an intervention to increase mobility in older patients: a qualitative study. J Health Organ Manag. 2021;35(9):140‐162. doi:10.1108/JHOM-02-2020-0049 33960175 PMC9251644

[42] Goodyear‐Smith F , Jackson C , Greenhalgh T . Co‐design and implementation research: challenges and solutions for ethics committees. BMC Med Ethics. 2015;16:78. doi:10.1186/s12910-015-0072-2 26573410 PMC4647576

[43] Connolly B , Milton‐Cole R , Adams C , et al. Recovery, rehabilitation and follow‐up services following critical illness: an updated UK national cross‐sectional survey and progress report. BMJ Open. 2021;11(10):e052214. doi:10.1136/bmjopen-2021-052214 PMC8491421 34607869

[44] Veloso Costa A , Padfield O , Elliott S , Hayden P . Improving patient diary use in intensive care: a quality improvement report. J Intensive Care Soc. 2021;22(1):27‐33. doi:10.1177/1751143719885295 33643429 PMC7890763

[45] Drewitz KP , Hasenpusch C , Bernardi C , et al. Piloting an ICU follow‐up clinic to improve health‐related quality of life in ICU survivors after a prolonged intensive care stay (PINA): feasibility of a pragmatic randomised controlled trial. BMC Anesthesiol. 2023;23(1):344. doi:10.1186/s12871-023-02255-1 37838669 PMC10576359

[46] Lasiter S , Oles SK , Mundell J , London S , Khan B . Critical care follow‐up clinics: a scoping review of interventions and outcomes. Clin Nurse Spec CNS. 2016;30(4):227‐237. doi:10.1097/NUR.0000000000000219 27309787 PMC4911825

[47] Duong J , Wang G , Lean G , Slobod D , Goldfarb M . Family‐centered interventions and patient outcomes in the adult intensive care unit: a systematic review of randomized controlled trials. J Crit Care. 2024;83:154829. doi:10.1016/j.jcrc.2024.154829 38759579

[48] Lange S , Mȩdrzycka‐Da Browska W , Friganović A , Religa D , Krupa S . Family experiences and attitudes toward care of ICU patients with delirium: a scoping review. Front Public Health. 2022;10:1060518. doi:10.3389/fpubh.2022.1060518 36505003 PMC9727388

[49] Schwartz AC , Dunn SE , Simon HFM , et al. Making family‐centered Care for Adults in the ICU a reality. Front Psychiatry. 2022;13:837708. doi:10.3389/fpsyt.2022.837708 35401268 PMC8987300

