# Supplementary material for: The implementation and evaluation of a family‐led novel intervention for delirium prevention and management in adult critically ill patients: A mixed‐methods pilot study
Source: Nurs Crit Care. 2024 Dec 1;30(4):e13210. doi: 10.1111/nicc.13210 (PMC12224221; doi:10.1111/nicc.13210)
Supplement: Supplementary file 1 — Data S1. Supporting information. [file NICC-30-0-s001.docx]

**Supplementary Materials**

**The FAMVR intervention messages**

TITLE: **The Development, Implementation and Evaluation of a Novel Intervention for Delirium in an Adult Intensive Care Unit: A Pilot Study**

**Domain 1 – General Reorientation to the ICU**

**Scripted Messages for ICU Reorientation** to be played 3 times a day (Morning, afternoon and bedtime)

**Please speak in a soft tone. These messages are a guide for you and you may use your own natural English words.**

**Introduction**

Hello/Hi/Hey **(Say patients name with any familiar or usual endearment or**

**word**). This is ---------- **(your name or endearment)** your **(your relationship to the**

**patient)**. I’m sending you this message to help you understand where you are and what is happening around you.

1. Your **nurse** is going to tell you the current date and time (The nurse will **insert time of the day, day of the week, date, month, year**).
2. Your **nurse** will also tell you how long you have been unwell for **(**The nurse will **insert length of time in ICU, illness, disease or accident that the patient is experiencing based on what you think would help)**.
3. You are in **(Insert name of the hospital).**
4. I know that this may be confusing, but you are being looked after in the hospital.
5. Your doctors and nurses care about you and are looking after you.
6. You might hear noises and beeps that are coming from the machines that you may or may not be able to see, but they are helping you to get better.
7. Please try to stay calm and relax **(endearment)** as the nurses and doctors

work to help you feel better.

1. I feel safe that you are in good hands here.
2. **(Specific family member or our family)** know you are here and have visited you

**(Past tense)** and thinking of you.

1. I Love You **(Usual endearment, closure or how you would usually end a conversation with them)**.

**Domain 2 – Specific ICU Routines**

**Scripted Messages for ICU Reorientation** to be played before routine nursing care as needed.

**Please speak in a soft tone. These messages are a guide for you and you may use your own natural English words.**

**Introduction**

Hello/Hi/Hey **(Say patients name with any familiar or usual endearment or**

**word**). This is ---------- **(your name or endearment)** your **(your relationship to the**

**patient)**. I’m sending you this message to help you understand where you are and what is happening around you.

1. Your **nurse** is going to tell you the current date and time (The nurse will **insert time of the day, day of the week, date, month, year**).
2. Your **nurse** will also tell you how long you have been unwell for **(**The nurse will **insert the length of time in ICU, illness, disease or accident that the patient is experiencing based on what you think would help)**.
3. You are in **(Insert name of the hospital).**
4. I know this may be confusing, but you are safe.
5. Your nurse is about to wash you in the bed and then clean your teeth, and will be rolling you to your side.
6. There are other nurses holding you gently to make sure you are safe.
7. You may be in some pain or feel strange but this is normal.
8. **(Specific family member name/all of our family)** know you are here and **(I/We)**

visited you and are thinking of you and looking after you too.

1. **(I/We)** are making sure the nurses and doctors know about you and how best to help you.
2. **(I/We)** Love You **(Usual endearment, closure or how you would usually end a conversation with them)**.

**Domain 3 – Specific ICU Procedures**

**Scripted Messages for ICU Reorientation** to be played before extubating/removing the breathing tube.

**Please speak in a soft tone. These messages are a guide for you and you may use your own natural English words.**

**Introduction**

Hello/Hi/Hey **(Say patients name with any familiar or usual endearment or**

**word**). This is ---------- **(your name or endearment)** your **(your relationship to the**

**patient)**. I’m sending you this message to help you understand where you are and what is happening around you.

1. Your **nurse** is going to tell you the current date and time (The nurse will **insert time of the day, day of the week, date, month, year**).
2. Your **nurse** will also tell you how long you have been unwell for **(**The nurse will **insert the length of time in ICU, insert illness, disease or accident that the patient is experiencing based on what you think would help)**.
3. You are in **(Insert name of the hospital).**
4. Could you open your eyes if you can?
5. I am not able to hear you because of the tube you have that is helping you to breath.
6. Is it possible you could do normal breathing then we can prepare to take this tube out of your mouth?
7. The nurses know you are uncomfortable and are doing everything they can to help you get better.
8. Please try to stay calm and relax **(endearment)** as the nurses and doctors

work to help you feel better.

1. **(Specific family member/All family member)** know you are here and visited you and thinking of you and talking to the nurses and doctors looking after you to make sure they know about you.
2. **(I/We)** love you/miss you/wish you the best **(Usual endearment, closure or how you would usually end a conversation with them)**.


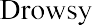

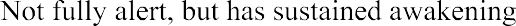

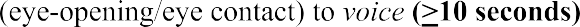

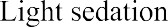

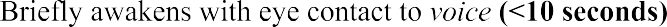

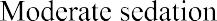

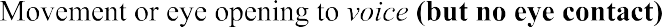

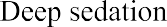

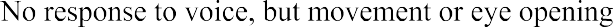

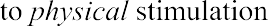

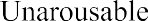

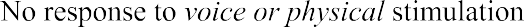

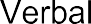

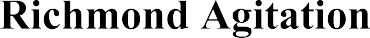

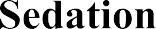

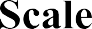

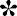


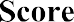

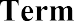

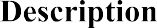

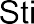

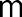

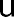

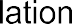

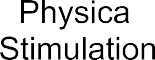


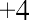

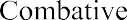

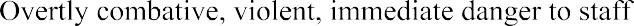

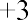

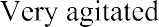

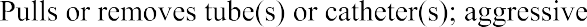

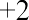

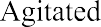

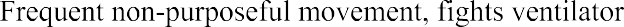

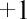

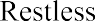

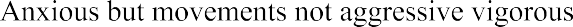

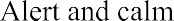


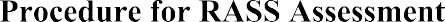

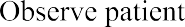

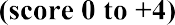

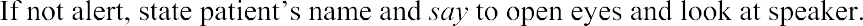

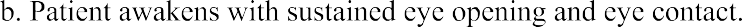

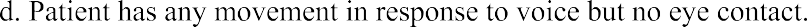

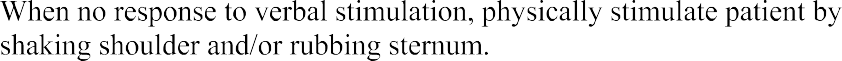

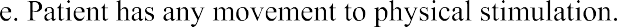


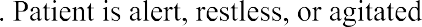

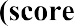

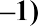

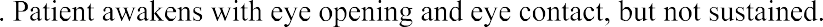

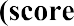

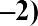

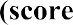

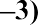

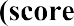

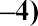

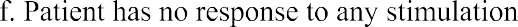

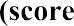

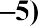


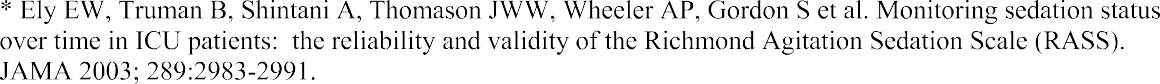


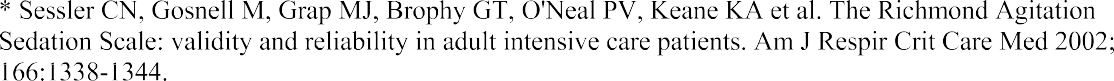


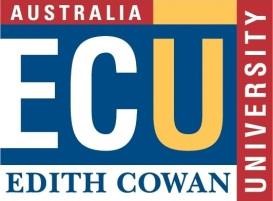


**Semi-Structured Interview Questions**

**Title: The Development, Implementation and Evaluation of a Novel Intervention for Delirium in an Adult Intensive Care Unit: A Pilot Study**

**Participants to be contacted via phone within four weeks from discharge.**

| **Participant Unique ID Number:** | |  | |
| --- | --- | --- | --- |
| **Date and time of interview** | |  | |
| **Date discharged from Hospital** | |  | |
| **How many people present for interview** | |  | |
| **Interview with patient** |  | **Interview with family**  **member/s** |  |
| **Relation of family member/s to**  **patient** | |  | |
| **Interviewee** | |  | |

**SWITCH RECORDER ON**

**Preamble**

Thank you for taking the time to participate in our study and your information is important to us. The interview will focus on you and/or your relative/friend’s experience whilst in hospital and after discharge with regards to the episode of delirium experienced by you or X. We are trying to

understand your experiences of the family member’s voice reorientation intervention that was provided to you or X, and how this intervention impacted you or X before discharge from the hospital.

**Questions**

1. Could you tell me about when you (or family member) were in intensive care and what happened to you?
2. Tell me about your experience on how your (or family member’s) delirium was prevented or managed in intensive care. (the interviewer will describe some of the key symptoms of delirium)

Probes: Interviewer to explore whether the diagnosis of delirium was shared with the patient and family or another term used to describe the experience.

1. Were you provided with any written or verbal information at admission or during your stay regarding delirium?
2. If so, what were you provided with?
3. Did you have any gaps in the information provided that would have been helpful?
4. Is there information or links to the FamVR provided to you?

Possible probes: Did you use the FamVR? Did you find the FamVR helpful? If so how did it help you?

1. Did you sourced additional information and support on delirium during your hospital stay and since being discharged? If so, what have they been?
2. Have you had the opportunity to speak about it with your general practitioner since being discharged? Did they provide you with any further support? Approximately how many times have you seen the GP?
3. Do you feel different from a mental perspective since you came out of hospital? Do you feel that you have returned to normal?

Probe: concentration, memory short/long term, irritability, "mixed up"

1. Have you (they) been readmitted into hospital since?
2. What medications are you currently taking? (Patient only)
3. Is there anything that else that you would like to say?

Thank you for participating in the interview.


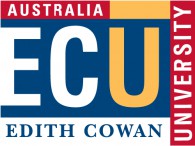


**Focus Group Interview Questions**

**Title: The Development, Implementation and Evaluation of a Novel Intervention for Delirium in an Adult Intensive Care Unit: A Pilot Study**

| **Participant Unique ID Number:** |  |
| --- | --- |
| **Date and time of focus group** |  |
| **Location** |  |
| **How many people present for focus group** |  |
| **Clinical Role** |  |
| **Interviewee** |  |

**SWITCH RECORDER ON**

**Preamble**

Thank you for taking the time to participate in our study and your information is important to us. The group interview will focus on your experience in intensive care with regards to the episode of delirium experienced by your patients. We are trying to understand your experiences of the family member’s voice reorientation intervention that was provided to your patients by you or your colleagues, and how this intervention impacted the patients before discharge from the intensive care unit.

**Questions**

1. Could you tell me about what happened to your patients when they were in intensive care and what happened to you?
2. Tell me about your experience on how your patients’ delirium was prevented or managed in intensive care. (The interviewer will describe some of the key symptoms of delirium) Probes: Interviewer to explore whether the diagnosis of delirium was shared with the patient and family, or another term used to describe the experience.
3. Did you provide your patients and relatives with any written or verbal information at admission or during their stay regarding delirium?
4. If so, what did you provide them with?
5. Did you have any gaps in the information provided that would have been helpful?
6. Is there information or links to the FamVR provided to them?

Possible probes: Did you use the FamVR? Did you find the FamVR helpful? If so, how did it help you care for your patients?

1. Did you source additional information and support on delirium while using the FamVR? If so, what have they been?
2. Have you had the opportunity to speak about delirium and the FamVR with your patients, relatives, and colleagues? Did they provide you with any further information or support? Approximately how many times did you use the FamVR?
3. Do you feel a difference in your mental health, that of your patients and relatives? Do you feel that you have returned to normal?

Probe: concentration, memory short/long term, irritability, "mixed up"

1. Have they been readmitted into hospital since?
2. Is there anything that else that you would like to say?

Thank you for participating in the interview.
